# Artificial Intelligence and Machine Learning Self-Assessment for Spinal Fusion Surgery: A Case Report

**DOI:** 10.7759/cureus.99848

**Published:** 2025-12-22

**Authors:** Ralph J Lamson

**Affiliations:** 1 Stanford Psychiatry Immersive Technology Consortium, Stanford University, Stanford, USA

**Keywords:** ai, logistic regression, mental wellbeing, path analysis, physical exercise, psycho-diagnostics & therapy, self-assessment tool, spinal fusion surgery, synthetic data

## Abstract

This is a report on self-assessment using Python, Artificial Intelligence (AI), and machine learning to predict patient readiness for spinal fusion surgery, including an analysis of whether the decision tree model recommended surgery.

The case of a 79-year-old retired psychologist (the author) with spinal stenosis, a collapsed L4-L5 disk, and crushed exit spinal nerves is explored. A boosted decision tree was used for prediction, supported by logistic regression and path analysis. Synthetic data were used alongside real patient data to add variability to the dataset. In this study, patient responses to a questionnaire were tested to determine if spine fusion surgery would be recommended. The results are limited by single-case and synthetic data. The model consists of a unique patient data array. Python, AI, and machine learning generated a self-assessment approach that offers patients and healthcare professionals an effective prediction tool.

Each year, a substantial number of patients ultimately require spinal surgery after experiencing prolonged or refractory back pain. Self-assessment is a tool for personal decision-making. It adds to a collaborative approach with healthcare providers. Wearable sensors to record spinal disk and nerve pain would be beneficial. In clinical practice, only a small proportion of healthcare AI research incorporates real-world patient data, with most studies relying on simulated or secondary datasets. The case demonstrates the efficacy of synthetic data in predictive modeling, while acknowledging the limitations in generalizing the findings to broader patient populations without real-world data.

## Introduction

Spinal fusion is a surgical procedure that involves joining two or more vertebrae together in order to stabilize the spine and alleviate lower back pain. The intervention is an option when motion is the source of pain, such as movement that occurs in a part of the spine that is arthritic or unstable due to injury, disease, or the normal ageing process. It is also of interest among medical professionals who conduct evaluations of the patient’s condition. Diagnostic X-rays, computed tomography (CT) scans, and magnetic resonance imaging (MRI) scans assist in the identification of the source of pain and site for intervention. An excellent source for spine medical terms and definitions is Mosby's dictionary, and a comprehensive history of spine surgery and its evolution has been published in Cureus medical research [1]. Spine history in a narrative form is presented by the authors, who cite early interventions categorized by time that include Egyptian and Babylonian, Greek and early Byzantine, Arabic, and Medieval periods. Reference to Hippocrates, Galen, and historical figures are mentioned in the publication of that manuscript, though a review here is beyond the scope of this study [1,2].

In one study, researchers deployed a statistical approach to investigate spinal fusion surgery. The logistic regression model was intended to predict 30-day readmission risk after lumbar spinal fusion surgery. The participants were selected from a large national database. The purpose of this study was to compare the length of stay, patient outcomes, and hospital costs between patients having surgery early in the week and later in the week. The authors of this research hypothesized that patients undergoing surgery later in the week would experience worse outcomes when compared to patients undergoing surgery early in the week. The study confirmed modifiable and non-modifiable risks by providing a large sample for lumbar-specific fusion. The researchers emphasized preoperative counselling for high-risk patients that included age, fitness, health, and comorbidity such as diabetes and obesity. The model demonstrated good predictive performance. Surgical factors like the number of levels fused and the operative time were also significant predictors. The authors concluded that the model could help identify high-risk patients for targeted interventions to reduce readmissions. The retrospective study from a single institution and the results may not be generalizable to a wider patient population [3].

Current medical research literature explores and reports on patient surgical interventions. The 79-year-old patient in this study was a recipient of bone from the lamina and the inferior articular process, and was saved for grafting. The patient's medical record documented procedure is detailed in the case discussion section. In this research, AI was used as a tool to generate patient self-assessment results. The patient benefited from informed decision-making while contributing to collaboration between the patient, physicians, and surgeons.

The primary objective of this study is the introduction of AI as a tool for self-assessment that would lead to a referral for spinal fusion surgery. Decision tree and statistical tests suggest AI will be a valuable contribution to patient lower back assessment and physician recommendations. 

The significance of this research is the rapid deployment of data that confirms a physician's assessment of a patient's lower back pain. It is a contribution to patient decision-making efficacy when experiencing lower back pain and when faced with the choice of spinal fusion surgery to alleviate pain. Medical professionals and the public are valued readers. The presentation of medical terms, procedures, and personal anecdotes adds texture to the case. Collaboration with real patients in decision-making is being advocated. Founders, skilled scientists and technologists engaged in advancing the development of medical assessments contribute to the production of artificial intelligence. Relevant to this study, vertical AI in medicine refers to artificial intelligence and machine learning specifically trained on curated medical data (e.g., electronic health records, medical images, case reports) to deliver high accuracy and relevant outcomes for targeted healthcare challenges such as spinal fusion surgery. 

## Case presentation

The case of a 79-year-old retired psychologist (the author) with spinal stenosis, a collapsed L4-L5 disk, and crushed exit spinal nerves is explored. A boosted decision tree was used for prediction, supported by logistic regression and Clopper-Pearson confidence interval. Synthetic data was used alongside real patient data to add variability to the dataset. In this study, patient responses to a questionnaire were tested to determine if spine fusion surgery would be recommended. The results are limited by single-case and synthetic data. The model consists of a unique patient data array. Python, AI, and machine learning generated a self-assessment approach that offers patients and healthcare professionals an effective prediction tool. Annually, approximately 450,000 patients undergo spinal surgery following prolonged back pain. Self-assessment is a tool for personal decision-making. It adds to a collaborative approach with healthcare providers. Wearable sensors to record spinal disk and nerve pain would be beneficial.

Procedure

Details of the procedure were obtained from the patient's medical record. The author, who was also the patient, grants permission to publish this research. 

Lateral fluoroscopy was utilized for localization of the operative site. A midline cutaneous incision was made from the spinous process of L3 to L5 using sharp dissection. Underlying subcutaneous subfascial plane dissection was performed using electrocautery until the spinous processes were identified. Only the right side of the fascia from L3 to L5 was exposed. A metal marker was placed under the lamina of L4. Intraoperative radiographs were obtained and confirmed with radiology to determine appropriate vertebral levels. Upon confirmation, dissection then proceeded laterally at the periosteal plane of the lamina to the lateral facet margin and further laterally with complete exposure at the base of the transverse processes at L4 to L5. Deep wound retractors were placed for operative exposure.

Attention was then directed towards the right L4 transverse process-lateral pedicle wall intersection. A pedicle screw was inserted using the standard technique. First, a high-speed bur was utilized to enter the center of the pedicle. Next, the gearshift probe was placed into the pedicle. The pedicle sound was used to confirm there was no cortical breach. Next, the hole was tapped and confirmation was again obtained, with a pedicle sound, that there were no pedicular breaches. All screws had excellent exertional torque. Screws were tested with an EMG probe. The pedicle screw was stimulated to greater than 20 milliamperes. This was repeated at L5 with similar results. All screws tested at greater than 20 milliamperes of current. Rod and set screws were placed. Compression force was then applied across the pedicle screw interval, and set screws were tightened to appropriate torque fixation. Intraoperative fluoroscopy verified the levels, position of the interbody, screw placement and overall alignment, kuo. 

AI, statistical logistic regression, and Clopper-Pearson confidence interval demonstrate associations of the patient questionnaire with outcome, surgery [2]. Linear regression cannot be used as a method of computation for referral to spinal fusion surgery. Linear regression assumes the target is continuous and unbounded. In comparison, logistic regression is designed specifically for binary outcomes. It models probability properly using the sigmoid function and correct statistical assumptions (Table 1).

**Table 1:** Comparison of linear with logistic regression. Logistic regression generates an output between 0 and 1.

Feature	Linear regression	Logistic regression
Outcome type	Continuous	Binary/categorical
Predicted values	Can be <0 or >1	Always between 0 and 1
Shape of relationship	Straight line	S-shaped (sigmoid)
Error distribution	Assumes normal	Assumes binomial
Estimation method	Ordinary least squares	Maximum likelihood
Suitable for probability?	No	Yes

It has been enlightening to investigate the cause and effect of lower back pain through personal history, assessment, diagnosis, treatment, and spinal fusion surgery. Patient reported that pre and post-surgery care has been influential in extending a healthy lifestyle. The study suggests that AI has the potential to integrate patients into therapeutic decision-making with data quality, model, and collaboration to improve diagnosis and treatment recommendations.

The lower back pain of the research patient intensified during 2021-2024. Before then, he enjoyed excellent health, improved mood, relaxation, and fitness. Muscle strain was thought to be the cause of pain. He applied ice, heat, stretched, meditated, swam, and supplemented these with over-the-counter medication. Each had a limited impact and duration for pain reduction. He experienced tingling and numbness in his right leg, including the three right toes of his right foot. At times, he felt as if he could fall.

**Figure 1:**
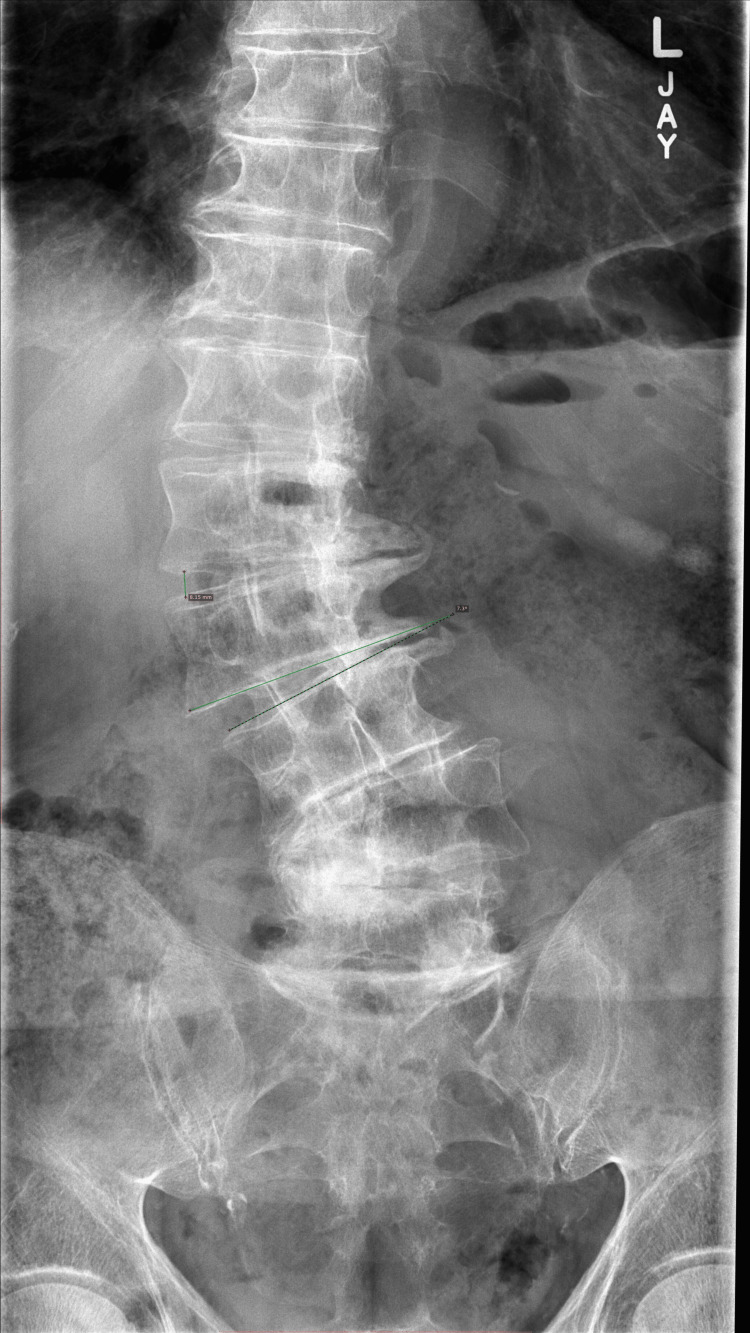
Spinal stenosis, L4-L5 disk bulge, neuroforaminal narrowing This radiograph shows spinal stenosis at the L4-L5 level, combined with disk bulge and neuroforaminal narrowing. Provider assessment of the condition indicated nerve openings (foramina) had become constricted as outlined in image. Age-related wear and disk damage have been cited in the literature as examples of nerve compression and symptoms like leg pain, numbness, tingling, and weakness.

**Figure 2:**
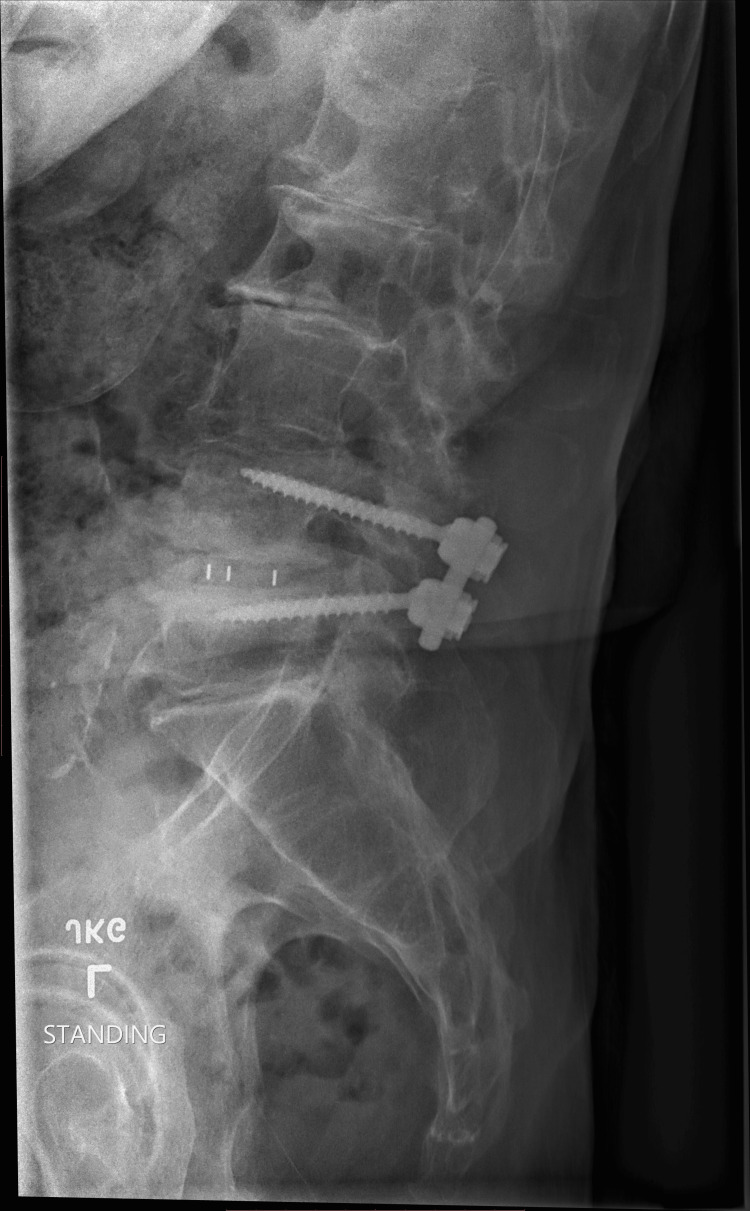
Post-spinal fusion surgery This radiograph shows spinal stenosis titanium hardware that was installed at the L4-L5 level during surgery.

Forty years of jogging contributed to the compression of a spinal disk located in L4-L5 and the crushing of existing spine nerves. His physician referred him for an MRI. Spinal stenosis and a compressed disk were diagnosed. Complementary approaches to pain reduction were recommended. The inability to attenuate pain led the patient to request an epidural. Pain decreased for 2.5 months and returned. He learned that pain was due to L4/L5 foraminal stenosis that affects the nerve roots that traverse at this level (Figures 1, 2). Faced with epidurals for the remaining life span, he accepted the recommendation for surgery. This case report was previously posted on preprints on 01 August 2025.

Motivation for conducting this research originated in 2024 after spine fusion surgery guided by curiosity. Computational skills utilized to generate data in this research are widely available. The paper contributes to patient self-assessment literature. It is unique in providing patients with a model for self-selected responses. The results are not generalizable, given the single case and synthetic data. However, imagine a site guiding users to construct their own questionnaire of 5 to 10 questions. Topics suitable for AI analysis include a range of applications such as mental health, addiction, physical distress, and those in combination. When numerical responses are entered, the data is entered into AI to generate results (Tables 2-5). 

**Table 2:** Research questionnaire key. Table of research questionnaire key features, questions and answers of real patient that were converted to read yes=1, no=0.

Key	Question	Response
Jogging	Does jogging contribute to lower back pain?	Yes
Time	How long have you been jogging?	40 years
Cause	What did you think caused your lower back pain?	Muscle strain
Evaluation	Did you receive a physician evaluation?	Yes
MRI	Did you get an MRI?	Yes
X-ray	Did you get an X-ray?	Yes
Diagnosis	Did you get a diagnosis?	Yes
What was your diagnosis	What was your diagnosis?	Stenosis of lumbar intervertebral foramina
Referral	Were you referred to physical medicine for an epidural?	Yes
Epidural	Did you get an epidural?	Yes
Surgery	Were you referred to physical medicine for L4-L5 minimally invasive surgery?	Yes
Consultation	Was the surgeon consultation kind, competent, and expert?	Yes
Disk	Did the consultation include a new plastic carbon fiber disk recommendation?	Yes
Titanium	Did the recommendation include one titanium rod with two screws?	Yes
Risks	Were you advised of the risks of surgery?	Yes
Recovery	Did the surgeon discuss recovery from surgery with you?	Yes
Fit	Is the patient physically and mentally fit?	Yes
What is your week: running, swimming, weight fitness?	What is your weekly routine: running, swimming, weight fitness?	The response is not specified in the input.
do you feel fit and healthy after 5 months post-surgery?	Do you feel fit and healthy after 5 months post-surgery?	Yes

**Table 3:** Self-assessment questionnaire conversion of responses. Feature or variable labels: There are eleven feature or variable labels that are represented by numbers in the above data array. For visual observation, this code introduces a list of feature labels that contains the names of each feature or variable. When calculating and printing the mean of each feature, it uses these labels to provide a readable output. The labels assist in understanding which feature each mean value corresponds to. feature_labels=("Jogging", "Time", "Evaluation", "Mri", "X-ray", "Referral", "Titanium", "Recovery", "Fit", "Lifetime Exercise", "Target").

Features or Variables	Responses
Jogging	1
Time	40
Evaluation	1
MRI	1
X-ray	1
Referral	1
Titanium	1
Recovery	1
Fit	1
Lifetime exercise	5
Target	1

**Table 4:** The patient data informs the synthetic dataset by modelling responses to questions as shown in this table. The synthetic data was generated after first real patient response.

Questions	Responses
Surgery (1=yes, 0=no)	1, 0, 1, 0, 0, 1, 1, 0, 0, 1
Titanium rod usage (1=yes, 0=no)	1, 0, 1, 0, 0, 1, 0, 0, 0, 1
MRI taken (1=yes, 0=no)	1, 0, 1, 0, 0, 1, 1, 1, 0, 1
Consultation quality (1=good, 0=poor)	1, 1, 0, 0, 1, 1, 1, 0, 0, 1
Recovery status (1=good, 0=poor)	0, 1, 1, 0, 0, 1, 1, 0, 0, 1
Patient’s fitness	1, 1, 1, 0, 1, 1, 1, 0, 0, 1
Outcome (1=positive, 0=negative)	1, 0, 1, 0, 0, 1, 1, 0, 0, 1

**Table 5:** Mean of features or variables. Understanding mean values: Each value represents the average of a particular feature (column) in the data set. If there were only one real patient (the first row), the remaining synthetic data must have been generated around this reference mean to simulate multiple records. The first row in the data array is real patient data. From there, the mean calculation for each feature is a direct reflection of the first row combined with the synthetic data. Role of synthetic data: To fill the data set, synthetic data introduced variability based on the data of the real patient (mean values). These synthetic rows simulate hypothetical patients with differing but similar attributes, allowing the data set to be expanded for analysis or machine learning training. Synthetic data is useful: Simulation of real-world scenarios: generating synthetic patient cases allows the model to understand the potential variability in real-world data. different years of jogging, varying levels of fitness, or cases where certain evaluations were not performed all contribute to a realistic training dataset.

Features or Variables	Mean
Mean of each feature	[0.6 18 0.6 0.6 0.6 0.6 0.4 0.6 0.4 3.6]
Mean of jogging	0.60
Mean of time	18.00
Mean of evaluation	0.60
Mean of MRI	0.60
Mean of X-ray	0.60
Mean of referral	0.60
Mean of titanium	0.40
Mean of recovery	0.60
Mean of fit	0.40
Mean of lifetime exercise	3.60
Mean of target	0.40
Proportion of 'surgery recommended'	0.4
Mean of evaluation	0.60
Mean of MRI	0.60
Mean of X-ray	0.60
Mean of referral	0.60
Mean of titanium	0.40
Mean of recovery	0.60
Mean of fit	0.40
Mean of lifetime exercise	3.60
Mean of target	0.40
Proportion of surgery recommended	0.4

The case demonstrates the efficacy of synthetic data in predictive modelling, while acknowledging the limitations in generalizing the findings to broader patient populations without real-world data.

## Discussion

AI produced results comparable with those of the recommended medical protocol. The questionnaire variables in this study were drawn from diagnostic imaging (MRI) and observed physical functioning gathered over the life course of the study participant. The contrast in time could not be greater. A decision tree is supervised learning employing labeled data that can be entered into AI. An individual’s health is influenced over time by physical challenges, human and natural influences, and education that come to occupy consciousness. To be clear, personal choices interact with real-world events, as did decades of jogging that likely contributed to spine nerve pain [4]. An individual may be the most informed expert by exercising self-efficacy over choices and behavior as suggested by Bandura [5]. The patient enjoyed excellent health until lower back pain worsened over time. The pain was misattributed to muscle strain. In a recent discussion that reflected on diagnostic decisions, some are influenced by initial biases, resulting in faulty conclusions, while decisions that take time are more likely to be the result of better information [6,7]. This study relates decades of patient experience, providers, and diagnosis to AI self-assessment.

Literature, complementary medicine, and motivation for research

Complementary medicine identifies approaches to pain reduction [8]. Meditation is an example of one approach that has the effect of pain reduction by distraction, cognitive restructuring, and a shift in perception. In contrast, virtual reality immersion therapy is a type of conventional treatment known as exposure therapy [9,10]. The therapy uses scientifically backed methods and is applied within a conventional medical context, unlike most complementary approaches. Virtual reality therapy is evidence-based. Unlike many complementary and alternative medicine (CAM) practices, virtual reality immersion therapy has been developed and studied using standard scientific research practices [8].

VR therapy is an interactive visual experience guided by a licensed therapist who has gained attention and applied it to a range of psychological stress-related conditions, such as phobias, social anxiety, and panic. The onset of distress and pain is documented during patient interviews, assessments, and interventions [9,10]. Virtual therapy is applied to reduce mental, emotional, and physical distress and is described in a USPTO patent. Additional VR applications were developed for substance-related conditions, cognitive impairment, and pain management, as stated in the meta-analysis report [11]. The list of applications has expanded since its introduction in the early 1990s. Among all diseases globally, mental illnesses are one of the major causes of burden. As many people are resistant to conventional evidence-based treatments, there is an unmet need for the implementation of novel mental health treatments. Efforts to increase the effectiveness and benefits of evidence-based psychotherapy in psychiatry have led to the emergence of virtual reality-based interventions. These interventions have shown a wide range of advantages over conventional psychotherapies. Currently, VR-based interventions have been developed mainly for anxiety-related disorders; however, they are also used for developmental disorders, severe mental disorders, and neurocognitive disorders.

Data presentation approach

Python coding and AI were skills learned at Stanford Code-in-Place, certificate awarded training using the Jupyter platform. Advanced AI training was undertaken and completed at Harvard on the GitHub platform and deeplearning.ai. These were followed by an online course in machine learning with PyCharm and Google AI [12-17]. 

Boosted decision tree XGBoost (see the decision tree figure in the supplemental section) results were achieved with boosting to emphasize incorrectly classified samples. Boosting produces higher accuracy for the case report self-assessment questionnaire dataset. Feature importance shows the influence of each feature or variable in predicting surgery. Solutions were inspired by or based on examples shown in class materials [18].

**Table 6:** Data presentation and analytical approach.

Method	Platform	Outcome	Key insight
AI, machine learning, decision tree with XGBoost (boosting)	Jupyter/PyCharm	Surgery recommendation	AI integration enables patient-centric therapeutic decision-making by combining data, modeling, and clinician collaboration.This approach contributes to diagnosis and treatment personalization.
Logistic regression	Jupyter/Colab	Surgery recommendation	Binomial logistic regression is used when the dependent variable has only two possible categories.
Clopper-Pearson confidence interval	Python	Surgery recommendation 100%	Exact binomial test valid for 5 study participants

Limitations

This study has several limitations. First, the study design includes a self-assessment questionnaire that has not been validated beyond the case. Therefore, it is suggested that a larger cohort of individuals be included in future research on spinal fusion surgery. A second limitation is the forced choice of variables entered into AI. If self-assessment is meant to be patient-centric, then a questionnaire coded to permit user preferences would be desirable. An obstacle remains. A patient-centric case would require synthetic data to generate a decision tree. The obstacle could be reduced by validation of features or variables most selected by a cohort of patients who have undergone spinal fusion surgery, and by those considering surgery. Demographic, physical functioning, and mental health information of the patient would be useful for physicians, surgeons, and patients when considering the treatment.

Future research

Numerical scales for assessment of pain associated with spinal stenosis of the lumbar spine rely upon patient report. The evaluation is a function of patient perceptions, given the variability of pain experience. Spine pain sensor detection specific to the location of L4-L5 may offer patient and provider real-time evaluation of pain during the assessment period of the condition [18]. The authors state that the ascending somatosensory pathways are responsible for conveying environmental and bodily sensory information from the peripheral organs to the central nervous system. Once established, will it be possible to monitor and record the transmission of signals with wearables? There is optimism. Stanford researchers report that overall, their new platform holds potential to yield insights into how the human sensory pathways assemble and therefore to develop and screen therapeutics for pain and other sensory system-related disorders. AI and machine learning contribute to the evolving history of patient self-assessment and spinal fusion surgery [19,20]. 

## Conclusions

Artificial intelligence (AI), machine learning, and decision tree of data from the patient self-assessment questionnaire reveal significant associations with the binary outcome of spinal fusion surgery. The patient's case history underscores the chain of events that led to lower back pain. These include a 40-year jogging history, physician assessment, MRI diagnosis, L4-L5 spine epidural treatment, and referral for spinal fusion surgery. The patient reports that structured pre and post-surgical care extends healthy lifestyle duration. AI integration enables patient-centric therapeutic decision-making by combining high-quality data, robust modeling, and clinician collaboration, potentially transforming diagnostic precision and treatment personalization.

The synthetic data array was generated to prepare the dataset for machine learning or statistical analysis, where a numerical format and sufficient diversity in samples are essential. This ensures that the model can be trained, tested, and perhaps be applied to broader real-world cases, even when only a single example is initially available. The synthetic data was generated around this single patient's data using the calculated mean values of each feature. This case report describes an experimental investigation assessing whether an artificial intelligence-based model could generate a decision tree to support referral or recommendation for spinal fusion surgery. The model successfully generated a decision framework, with additional support for surgical referral provided by logistic regression analysis and an exact binomial test using the Clopper-Pearson confidence interval.

## Figures and Tables

**Figure 3:**
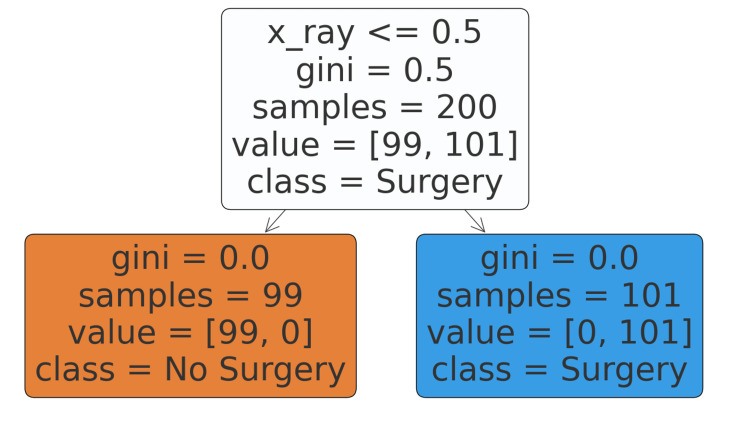
Decision tree. Summary of findings*: *DecisionTreeClassifier(max_depth=2, random_state=0). Max_depth=2 ensures readability. Reason for the Splits (Interpretation): all trees with the current data have a similar interpretation because of the underlying probabilities. Two-Split Decision Tree (Simple). Interpretation of the 2-Split Tree Split 1: x_ray. Was an X-ray ordered? This is the strongest indicator in your dataset. When real patients are evaluated in clinical settings, X-rays are often ordered when structural spinal issues are suspected. If x_ray = 1 (yes). The model predicts a high likelihood of surgery. This implies that imaging showed something serious, or the physician already had a high suspicion before imaging. This branch becomes Leaf C. Split 2 (only for x_ray = 0): evaluation If no X-ray was ordered, the next best signal is the clinical evaluation result. Was the initial evaluation positive / concerning? If evaluation=0 (normal/negative), low likelihood of spinal fusion (Leaf A). Patients are functioning well; no immediate red flags. If evaluation = 1 (positive/abnormal) Moderate chance of surgery (Leaf B) There are clinical concerns, but not yet enough to order imaging. Summary of Final Logic: Why this is a Good 2-Split Model. It uses the two strongest clinical variables, captures most of the signal from the full tree, easy for clinicians to interpret, Avoids overfitting, Produces three clear clinical pathways. Overall Behavior of the Tree The decision tree mainly follows clinically intuitive logic: Was there a clinical evaluation recommending surgery? If yes: more tests increase probability. Were MRI/X-ray/Referral positive? These dramatically raise likelihood of surgery. How physically active has the patient been? Higher lifetime exercise lowers probability because of presumed fitness. That conclusion conflicts with the real patient experience whose 40 years of jogging contributed to lower back pain that eventually required spinal fusion surgery.

**Figure 4:**
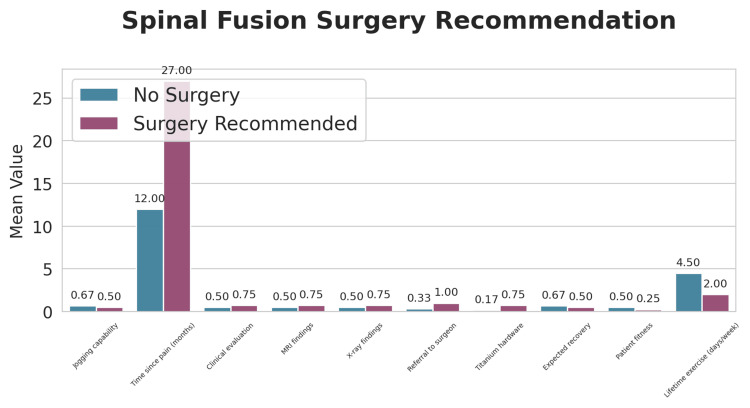
Logistic regression conditional means Predicted probability of surgery is produced from the logistic regression model.

**Figure 5:**
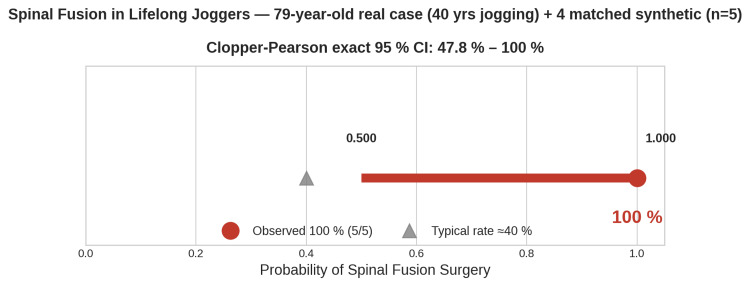
Exact binomial test of a Clopper-Pearson confidence interval The Clopper-Pearson confidence interval produced results for a highly homogeneous group of five dedicated joggers, one real 79-year-old man who jogged regularly for 40 years and four age-matched synthetic controls, all five required spinal fusion surgery.

## Appendices

Appendix A 

Appendix B 

Appendix C 

**Table 7:** Predicted probability of surgery is produced from the logistic regression model.

Model’s decision (threshold = 0.5).
The model uses the standard 0.5 cutoff: ≥0.5 to predict surgery.
Different stories are useful:
The bar graph of figure 4 illustrates: On average, how patients who end up getting surgery differ from those who don’t.
Table 8 data shows: How well the logistic model predicts patients who will actually get surgery.

Appendix D 

**Table 8:** Logistic regression.

Patient	True surgery	Predicted prob	Model’s decision (if threshold = 0.5)	Correct
0	1	0.8104	Predict surgery	Correct
1	0	0.2405	Predict no surgery	Correct
2	1	0.8145	Predict surgery	Correct
3	0	0.1402	Predict no surgery	Correct
4	0	0.2191	Predict no surgery	Correct
5	1	0.8283	Predict surgery	Correct
6	0	0.1402	Predict no surgery	Correct
7	1	0.8104	Predict surgery	Correct
8	0	0.1682	Predict no surgery	Correct
9	1	0.8283	Predict surgery	Correct

Appendix E 

**Table 9:** Predicted Probabilities Key Visual Insights.

"Surgery Recommended" (target: 0 = No, 1 = Yes).
The overall proportion of surgery recommended is 0.4 (40% Yes, 60% No). Conditional means for the two groups are illustrated in the figure to show the meaningful differences.
Patients recommended for spinal fusion surgery have much longer pain duration (27 vs 12 months).
Referral to surgeon is almost deterministic (100% when surgery is recommended).
Titanium hardware is planned in 75% of surgery cases vs only 17% when not recommended.
Surgery-recommended patients tend to have worse imaging findings (MRI/X-ray 0.75 vs 0.50).
They are less fit and exercise much less lifetime (2.0 vs 4.5 days/week).

Appendix F 

**Table 10:** Clopper-Pearson method, the 95% confidence interval for the true surgery rate in this cohort of 79 year old study participants is 47.8% to 100%. Clopper-Peason test in this experimental study adds support in the prediction that older patients with decades of regular running and fully completed diagnostic/referral pathways are at extremely high risk of needing lumbar spinal fusion.

Patient	Age	Years jogging	Eval	MRI	X-ray	Referral	Titanium	Recovery	Fit	Lifetime Exercise Score	Surgery
Real (79 yo)	79	40	1	1	1	1	1	1	1	5	1
Syn. 1	38	38	1	1	1	1	1	1	1	4	1
Syn. 2	42	42	1	1	1	1	1	1	1	6	1
Syn. 3	41	41	1	1	1	1	1	1	1	5	1
Syn. 4	39	39	1	1	1	1	1	1	1	5	1

Appendix G 

Pre-op diagnosis codes: Degenerative scoliosis (m41.50), osseous and subluxation stenosis of lumbar intervertebral foramina (m99.63), spinal stenosis of lumbar spine (m48.061).
